# Genome-Wide Association Mapping of Starch Pasting Properties in Maize Using Single-Locus and Multi-Locus Models

**DOI:** 10.3389/fpls.2018.01311

**Published:** 2018-09-05

**Authors:** Yang Xu, Tiantian Yang, Yao Zhou, Shuangyi Yin, Pengcheng Li, Jun Liu, Shuhui Xu, Zefeng Yang, Chenwu Xu

**Affiliations:** Key Laboratory of Crop Genetics and Physiology of Jiangsu Province, Key Laboratory of Plant Functional Genomics of Ministry of Education, Co-Innovation Center for Modern Production Technology of Grain Crops, Yangzhou University, Yangzhou, China

**Keywords:** maize, starch, pasting properties, GWAS, multi-locus

## Abstract

Maize starch plays a critical role in food processing and industrial application. The pasting properties, the most important starch characteristics, have enormous influence on fabrication property, flavor characteristics, storage, cooking, and baking. Understanding the genetic basis of starch pasting properties will be beneficial for manipulation of starch properties for a given purpose. Genome-wide association studies (GWAS) are becoming a powerful tool for dissecting the complex traits. Here, we carried out GWAS for seven pasting properties of maize starch with a panel of 230 inbred lines and 145,232 SNPs using one single-locus method, genome-wide efficient mixed model association (GEMMA), and three multi-locus methods, FASTmrEMMA, FarmCPU, and LASSO. We totally identified 60 quantitative trait nucleotides (QTNs) for starch pasting properties with these four GWAS methods. FASTmrEMMA detected the most QTNs (29), followed by FarmCPU (19) and LASSO (12), GEMMA detected the least QTNs (7). Of these QTNs, seven QTNs were identified by more than one method simultaneously. We further investigated locations of these significantly associated QTNs for possible candidate genes. These candidate genes and significant QTNs provide the guidance for further understanding of molecular mechanisms of starch pasting properties. We also compared the statistical powers and Type I errors of the four GWAS methods using Monte Carlo simulations. The results suggest that the multi-locus method is more powerful than the single-locus method and a combination of these multi-locus methods could help improve the detection power of GWAS.

## Introduction

Maize (*Zea mays* L.) is the world’s most important crop for food, feed and industrial materials. Starch is the principal constituent of maize kernels, which accounts for approximately 70% of the kernel weight (Liu N. et al., 2016). Benefitting from its characteristics such as slow tendency of retrogradation and low pasting temperature (PTP), maize starch serves as an essential ingredient for industrial production of food, and has been widely used to thicken sauces or soups and make corn syrup and other sugars (Yang Z. et al., 2014). Recently, great progress has been made in dissection of starch content in maize kernels (Wang et al., 2015; Li et al., 2018). However, further improvements in starch quality are needed to meet demands of food processing and industrial application. The pasting properties are important characteristics of starch, determining the starch quality and functionality. Dissection the genetic basis of pasting properties will facilitate the improvement of starch quality in maize.

Genome-wide association studies (GWAS) provide the opportunity to decipher genetic architectures of complex traits in crops (Zhu et al., 2008). Owing to the rapid linkage disequilibrium (LD) decay and abundant diversity, maize is an ideal species to perform GWAS. GWAS have successfully analyzed many important traits, such as kernel oil biosynthesis, plant height and disease resistance in maize (Kump et al., 2011; Li et al., 2013). Some statistical models have been developed to conduct GWAS. Mixed linear model (MLM) has become the most popular approach with the ability to consider population structure and family relatedness (Zhang et al., 2005; Yu et al., 2006). Based on the MLM framework, some single-locus approaches have been proposed to alleviate the heavy computational burden, such as EMMAX (Kang et al., 2008), P3D (Zhang et al., 2010), FaST-LMM (Lippert et al., 2011), and genome-wide efficient mixed model association (GEMMA) (Zhou and Stephens, 2012). However, the single-locus model testing one locus at a time fails to match the true genetic model of complex traits that are controlled by numerous loci simultaneously. Additionally, multiple test corrections for critical values are usually required to control false positive rates for single-locus GWAS. The commonly used Bonferroni correction is so conservative that lots of true loci may be neglected. To overcome these problems, multi-locus GWAS methods have been recommended because these methods consider the information of all loci simultaneously and multiple test corrections are not required because of the multi-locus nature (Wang et al., 2016). Several multi-locus methods, such as FASTmrEMMA, ISIS EM-BLASSO, FASTmrMLM, pLARmEB, pKWmEB, LASSO, and FarmCPU, have been proved to be more powerful than single-locus methods (Liu X.L. et al., 2016; Tamba et al., 2017; Xu et al., 2017; Zhang et al., 2017; Ren et al., 2018; Wen et al., 2018).

There have been a few studies focusing on the genetic basis of pasting properties in maize starch. Zhang et al. (2004) suggested that *SSIIa* of maize affected the starch structure and physiochemical properties. Wilson et al. (2004) used association mapping to evaluate six candidate genes involved in starch synthesis and found that *ae1* and *sh*2 were associated with starch pasting properties. Xu et al. (2014a) detected the associations of sequence variants of the *ZmBT1* gene with seven pasting properties. Yang Z. et al. (2014) identified seven quantitative trait nucleotides (QTNs) in coding regions of *Zmisa2* underlying pasting properties of maize starch and proposed that these markers may be potentially utilized for marker-assisted selection. However, all of the above studies were based on specific candidate genes involved in kernel starch biosynthesis. Therefore, more comprehensive studies are required to further understand the molecular mechanisms of starch pasting properties. To our knowledge, GWAS for pasting properties of maize starch have not been reported up to now.

In this study, a worldwide collection of 230 inbred lines were genotyped with 145,232 SNPs using genotyping-by-sequencing (GBS) technology. Starch pasting properties including peak viscosity (PV), trough viscosity (TV), final viscosity (FV), breakdown viscosity (BD), setback viscosity (SB), pasting time (PT), and PTP were measured for the 230 lines using the Rapid Visco Analyser (RVA). The main objectives of this study were to (i) identify loci that are significantly associated with pasting properties of maize starch using single-locus and multi-locus GWAS methods, and (ii) compare three multi-locus methods (FASTmrEMMA, LASSO, and FarmCPU) with one single-locus method (GEMMA) in terms of their detection powers and Type I errors.

## Materials and Methods

### Plant Materials

In this study, an association panel of 230 maize lines collected from the tropical, subtropical or temperate zone, representing a wide range of diversity, was used for GWAS. All the materials were planted with a randomized block design of three repetitions in the field of Sanya, Hainan province. At the four-leaf stage, young leave tissues were collected from each line and preserved at −80°C. DNA was extracted from the freeze-dried leave tissues with the modified CTAB method (Fulton et al., 1995). After harvest, mature kernels of five randomly selected plants in each line were collected and used for evaluation of starch pasting properties.

### Genotyping

The panel of 230 maize inbred lines was genotyped using a GBS strategy. The ApeK1 restriction enzyme was used for library preparation, and GBS was performed on an Illumina platform by Novogene Bioinformatics Institute, Beijing, China. After quality control, a total of 145,232 high-quality SNPs with minor allele frequency (MAF) above 2% and missing rate below 20% remained to perform GWAS.

### Measurement of Starch Pasting Properties

The pasting properties of maize starch were evaluated using RVA (Model 3D, Newport Scientific, Sydney, NSW, Australia). Three grams of starch obtained from each line was mixed with 25 ml of distilled water in the RVA canister. The RVA profile took a heat–hold–cool temperature cycle as follows: (1) set at 50°C as the starting temperature and maintained for 1 min; (2) heated to 95°C and held at 95°C for 2.5 min; and (3) cooled to 50°C and kept at 50°C for 1.4 min. The total processing time was about 12 min. The pasting properties were determined using a fixed paddle rotation at the speed of 160 r/m. The RVA parameters were recorded in centipose (cP). The pasting parameters obtained from the pasting curve including PV, TV, FV, PTP, PT and their derived parameters, BD and SB were recorded for all the inbred lines. The average value of three biological replicates from each line was obtained for data analysis.

### Genome-Wide Association Analysis

In this study, GWAS were performed in the association panel composed of 230 diverse maize inbred lines with 145,232 high-quality SNPs. The decay distance of LD across the whole genome was determined by software PopLDdecay^1^. Principle component analysis (PCA) was used to control for population structure. Both single-locus and multi-locus methods were used to identify significant QTNs for seven starch properties. GEMMA was used for single-locus GWAS, and FASTmrEMMA (Wen et al., 2018), LASSO (Xu et al., 2017), and FarmCPU (Liu X.L. et al., 2016) were used for multi-locus GWAS. GEMMA was developed based on the framework of MLM, which takes advantage of eigen decomposition to substantially increase the computational speed. GEMMA was implemented in the software GEMMA. FASTmrEMMA is a multi-locus two-stage GWAS method, combining the MLM and the expectation and maximization empirical Bayes (EMEB) method. In the first stage, the marker effects were treated as random and a small number of markers were selected, and then in the second stage, the selected markers were fitted into a multi-locus model and estimated using the EMEB method. FASTmrEMMA was implemented in the R package mrMLM. LASSO is a powerful multi-locus approach, but it lacks a default method to perform a significance test. Here, we used our previously proposed Bayesian algorithm to approximately estimate the variance of each marker effect and then used a Wald test to obtain the significant test for each marker. Details about this algorithm were given in Xu et al. (2017). The LASSO method was implemented in the R package glmnet and our own R program. The FarmCPU method is a commonly used GWAS method at present, which effectively eliminates confounding and improves statistical power for MLM methods by using the fixed effect model and random effect model iteratively. FarmCPU was implemented in the R package FarmCPU. All parameters were set at default values. The significantly associated QTNs were determined by the LOD value exceeding three for FASTmrEMMA and LASSO, and the *P*-value less than 1/*m* (*m* is the number of markers) for GEMMA and FarmCPU. To mine candidate genes based on the detected QTNs for the pasting properties, we used gene annotation and ontology information available in maizeGDB^2^ and Phytozome database^3^.

### Simulation Experiments

To investigate the powers and Type I errors of the single-locus and multi-locus GWAS methods, we carried out a Monte Carlo simulation experiment using the genotypic data of 230 maize inbred lines. We assigned eight QTL located on the first eight chromosomes. The assigned QTL totally explained 56% of the phenotypic variation. The detailed description of the eight QTL is presented in **Table 1**. Both the polygenic variance and residual error variance were set at one. The population structure effect was added according to the first five principal components determined from the genotypic data. These principal components contributed to 10% of the phenotypic variance. The phenotype was simulated with the contribution of the genetic effect of simulated QTL, polygenic effect, residual effect, and population structure effect. The simulations were replicated 200 times and the four GWAS methods, FASTmrEMMA, FarmCPU, LASSO, and GEMMA, had been used to analyze the simulated data. The statistical power for a simulated QTL was defined as the fraction of the 200 replicates where the LOD score of the QTL was larger than three for the FASTmrEMMA and LASSO methods and the *P*-value of the QTL was less than 1/*m* for GEMMA and FarmCPU. Type I error was defined as the ratio of false positives out of all markers not assigned a QTL effect. Each QTL within 1 kb of the assigned QTL was counted as a real QTL.

**Table 1:** Information for the eight simulated QTL.

QTL	Chromosome	Position (bp)	MAF	Effect	*R*^2,a^ (%)
QTL1	1	14898058	0.335	0.569	4
QTL2	2	19326559	0.16	0.842	4
QTL3	3	20532172	0.307	1.041	6
QTL4	4	13181343	0.452	0.667	6
QTL5	5	15819352	0.204	0.935	8
QTL6	6	27154881	0.378	0.766	8
QTL7	7	16672999	0.085	1.564	10
QTL8	8	22685122	0.217	1.028	10

*^a^Proportion of the total phenotypic variation explained by the QTL.*

## Results

### Phenotypic Variations and Heritability

**FIGURE 1:**
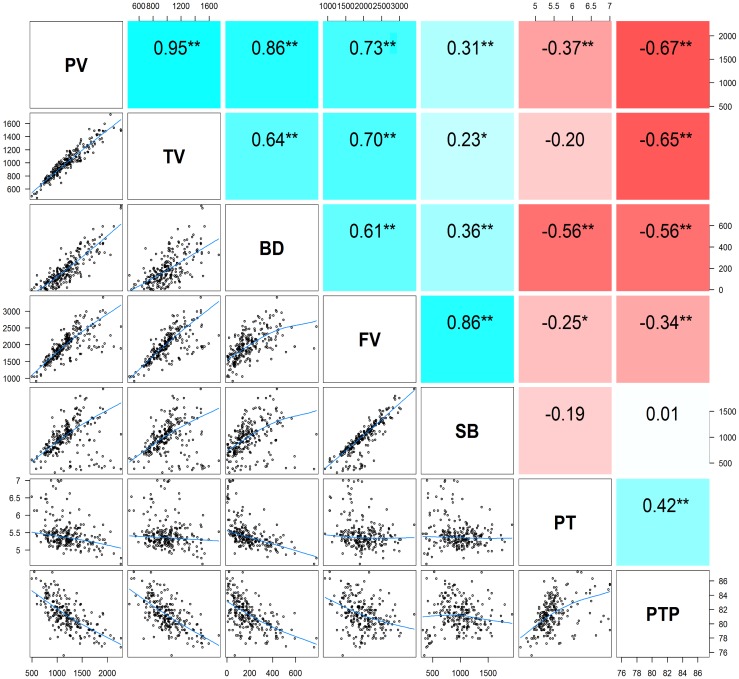
The pairwise correlation analysis among seven pasting properties of maize starch. *Upper diagonal*: Pearson correlation coefficients between every two traits; *Lower diagonal*: Scatter plots of correlations between every two traits. *Asterisk* (^∗^) indicates significance level at *P* < 0.05; *Double* *asterisks* (^∗∗^) indicates significance level at *P* < 0.01. PV, peak viscosity; TV, trough viscosity; BD, breakdown viscosity; FV, final viscosity; SB, setback viscosity; PT, pasting time; PTP, pasting temperature.

The descriptive statistics of the seven pasting properties for the 230 maize inbred lines are listed in **Table 2**. The average values for PV, TV, BD, FV, SB, PT, and PTP are 1,200.22, 1,004.04, 196.18, 1,980.36, 976.33, 5.46, and 81.24 with the standard deviations 334.56, 225.29, 141.77, 427.17, 314.40, 0.42, and 2.14, respectively. Substantial variations among genotypes are observed for the seven pasting properties, and pasting properties vary significantly among different lines. Also, variance components were estimated using the restricted maximum likelihood (REML) analysis (Xu et al., 2014b). The narrow sense heritability, defined as the ratio of additive genetic variance to total phenotypic variance, ranges from 0.46 for PT to 0.77 for TV (**Table 2**). These results indicate that the phenotypic variations of starch pasting properties are mainly affected by genetic factors, and therefore this panel can be used for further genetic analyses. To determine the correlation among the seven pasting properties, the Pearson’s correlation coefficients were calculated. The results of the correlation analysis are illustrated in **Figure 1**. All the pairwise correlations between any two pasting properties exhibit significantly positive or negative correlations except three correlations between PT and TV, between PT and SB, and between SB and PTP.

**Table 2:** Phenotypic performance, variance component, and heritability of seven pasting properties of maize starch.

	Mean ± SD	Range	Genetic variance	Residual variance	Heritability	*F* value
PV (cP)	1,200.22 ± 334.56	494.5–2,272	115,459.13	50,143.19	0.70	2.11^∗∗^
TV (cP)	1,004.04 ± 225.29	463.5–1,737	63,071.08	18,425.03	0.77	2.28^∗∗^
BD (cP)	196.18 ± 141.77	2.5–783.5	15,269.62	11,891.91	0.56	1.96^∗∗^
FV (cP)	1980.36 ± 427.17	920–3411	155519.10	99828.04	0.61	2.12^∗∗^
SB (cP)	976.33 ± 314.40	319–1930	108176.00	41637.66	0.72	2.40^∗∗^
PT (min)	5.46 ± 0.42	4.6–7	0.11	0.13	0.46	3.26^∗∗^
PTP (°C)	81.24 ± 2.14	75.55–87.28	3.40	2.86	0.54	2.71^∗∗^

*^∗∗^Indicates significance level at P < 0.01. PV, peak viscosity; TV, trough viscosity; BD, breakdown viscosity; FV, final viscosity; SB, setback viscosity; PT, pasting time; PTP, pasting temperature.*

### Population Structure and Linkage Disequilibrium

**FIGURE 2:**
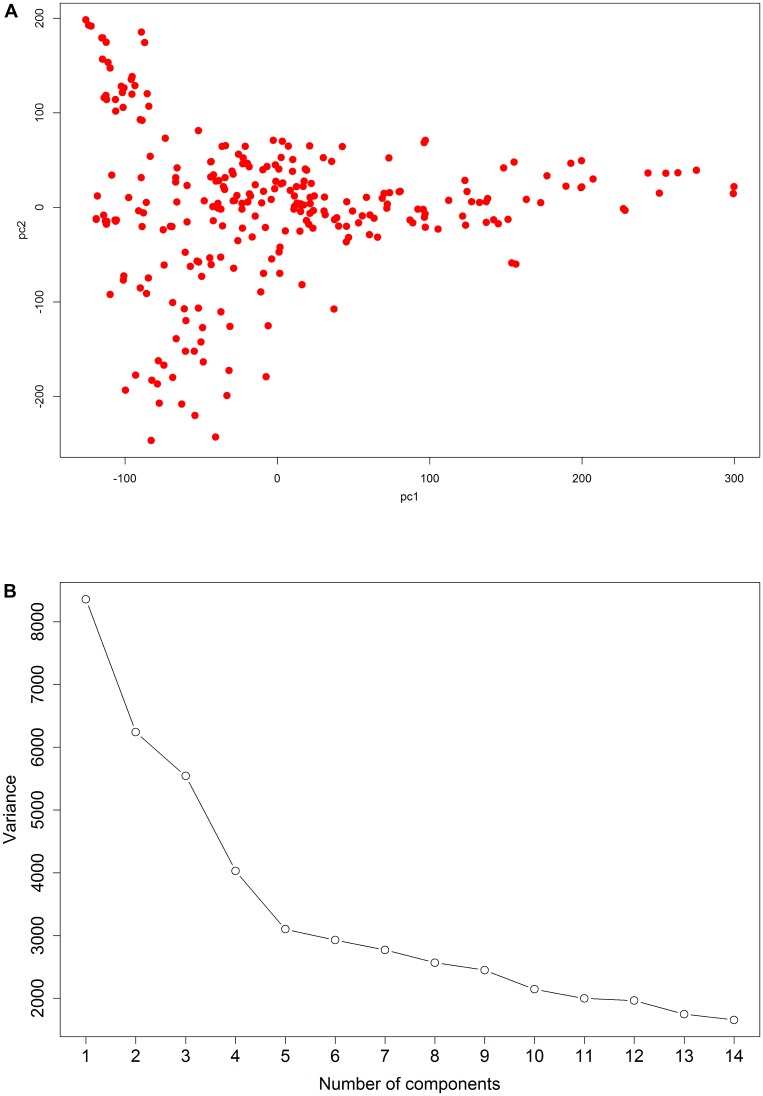
Genetic structure of maize inbred lines. **(A)** Plot of the first two principal components of 230 inbred lines. **(B)** Scree plot showing the selection of principal components for GWAS.

**FIGURE 3:**
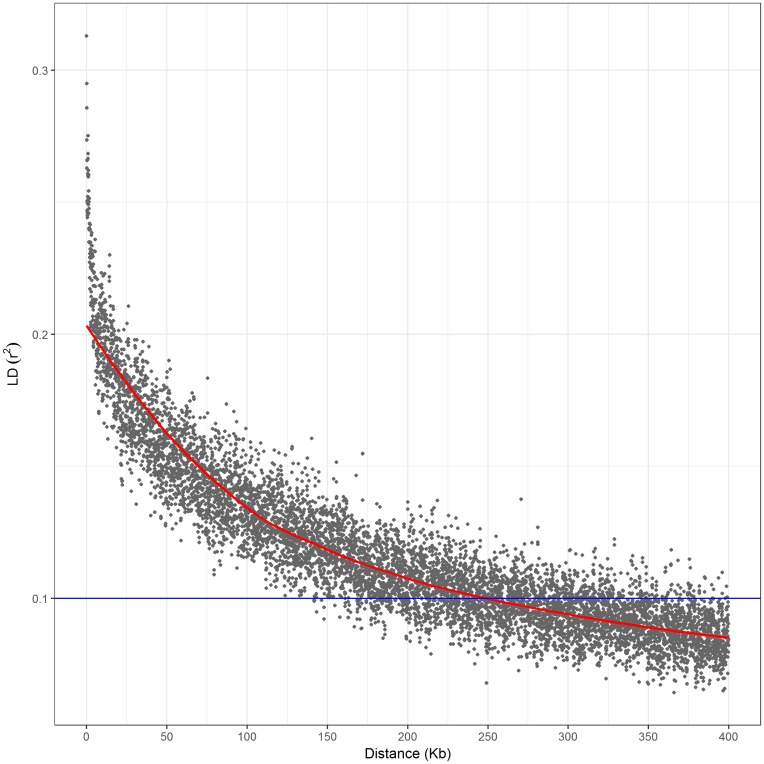
Linkage disequilibrium decay across the whole genome of the association panel. The *blue horizontal line* shows the LD threshold for the association panel (*r*^2^ = 0.1).

In this study, PCA was used to correct for population structure. PCA plots of this association population are illustrated in **Figure 2**. According to the scree plot, the variance of principle component score decreases quickly until the fifth principle component (**Figure 2B**). Therefore, we selected the first five principal components to control the population structure. All filtered SNPs were used to determine LD decay. A monotonic decrease in LD is found with increasing distance (**Figure 3**). At *r*^2^ = 0.2, the overall LD decay decreases dramatically to 10 kb. The genome-wide LD decay distance is about 250 kb at the cut-off of *r*^2^ = 0.1.

### GWAS for Starch Pasting Properties

In this study, GWAS were conducted for 230 maize inbred lines with 145,232 SNPs using four methods and the results are listed in **Table 3**. A total of 60 significant QTNs are identified for seven starch properties from the four GWAS methods. FASTmrEMMA detects the most QTNs (29), followed by FarmCPU (19) and LASSO (12), GEMMA detected the least QTNs (7). The numbers of significant QTNs detected for starch properties PV, TV, BD, FV, SB, PT, and PTP are 14, 10, 8, 12, 12, 6, and 5, respectively, from all the four methods. The corresponding numbers of the significant QTNs are 8, 6, 5, 3, 6, 1, and 4 from FASTmrEMMA; 2, 4, 2, 7, 4, 2, and 1 from FarmCPU; 2, 2, 0, 1, 3, 3, and 1 from LASSO; and 2, 0, 1, 1, 1, 2, and 0 from GEMMA. The largest QTN detected by FASTmrEMMA, FarmCPU, LASSO, and GEMMA explains 9.35, 14.96, 1.03, and 12.03(%) of the phenotypic variation, respectively. Among these significant QTNs, seven QTNs appear to control more than one trait (pleiotropic effect). For example, three QTNs (SNP_2_190495578, SNP_9_138239683, and SNP_4_89429269) have significant effects on PV and TV. Both SNP_6_109456130 and SNP_7_160060597 are associated with PV and BD. The correlations between PV and TV as well as between PV and BD are significant.

**Table 3:** Significantly associated QTNs identified by four GWAS methods for seven pasting properties of maize starch.

Trait	Marker	Alleles	Chr	Pos	FASTmrEMMA		FarmCPU		LASSO		GEMMA	
					Effect	*R*^2^ (%)	Effect	*R*^2^ (%)	Effect	*R*^2^ (%)	Effect	*R*^2^ (%)
PV	SNP_2_190495578^#^	C/A	2	190495578	256.70	7.05						
	SNP_2_42359599	A/G	2	42359599	−131.53	2.87						
	SNP_2_51001688	A/C	2	51001688	−191.54	5.03						
	SNP_3_171824570^#^	G/T	3	171824570	126.17	2.46						
	SNP_4_64845133	A/G	4	64845133	193.04	2.81						
	SNP_4_89429269^#^	G/A	4	89429269	134.34	3.09						
	SNP_5_26160368	T/A	5	26160368							−234.93	11.13
	SNP_5_26160478	C/A	5	26160478							−234.29	11.64
	SNP_6_109456130^#^	A/C	6	109456130			−116.84	7.12				
	SNP_6_164038368	C/A	6	164038368					−51.80	0.46		
	SNP_7_160060597^#^	A/G	7	160060597	174.52	2.79						
	SNP_8_147208913	C/T	8	147208913			−133.38	5.05				
	SNP_9_138239683^#^	C/A	9	138239683					−68.33	1.03		
	SNP_9_58569771	C/T	9	58569771	210.21	3.01						
TV	SNP_2_190495578^#^	C/A	2	190495578	148.11	5.18						
	SNP_2_75175274	A/G	2	75175274	−163.45	3.19						
	SNP_4_144401228^∗^	A/G	4	144401228	−141.31	4.68			−30.63	0.88		
	SNP_4_89429269^#∗^	G/A	4	89429269	103.79	4.07	54.80	4.56				
	SNP_5_168661067	G/C	5	168661067			−76.75	4.19				
	SNP_8_104430223	T/C	8	104430223	163.65	7.19						
	SNP_9_138239602	G/C	9	138239602			−102.16	4.94				
	SNP_9_138239683^#^	C/A	9	138239683					−44.03	0.94		
	SNP_10_12091187	A/G	10	12091187	−103.99	3.72						
	SNP_10_142948941	A/G	10	142948941			48.76	3.85				
BD	SNP_1_241610826	C/T	1	241610826			−33.08	4.33				
	SNP_1_825561	C/T	1	825561	93.47	3.56						
	SNP_4_146006182	G/A	4	146006182	73.78	3.74						
	SNP_6_109456130^#^	A/C	6	109456130			−58.28	9.86				
	SNP_7_160060597^#^	A/G	7	160060597	76.06	2.95						
	SNP_9_142242612	C/T	9	142242612	−49.54	2.53						
	SNP_10_138051694	G/C	10	138051694							82.38	10.28
	SNP_10_9143566	G/T	10	9143566	−83.04	5.87						
FV	SNP_1_283390691	T/C	1	283390691			106.83	5.53				
	SNP_2_51001706	C/T	2	51001706	−273.07	6.02						
	SNP_5_160490300	A/G	5	160490300			−142.99	5.16				
	SNP_5_160866262	T/C	5	160866262			167.30	6.97				
	SNP_5_213796937	A/G	5	213796937			−96.09	3.71				
	SNP_6_107223456	G/C	6	107223456							−206.80	12.03
	SNP_6_115373488	G/A	6	115373488	175.34	3.14						
	SNP_7_173235732^#^	T/G	7	173235732			130.23	5.04				
	SNP_8_124259102	A/C	8	124259102					−64.27	0.51		
	SNP_8_154309867	G/T	8	154309867			150.77	6.61				
	SNP_9_113510544	G/A	9	113510544	264.83	3.48						
	SNP_9_83760699	A/T	9	83760699			143.42	3.60				
SB	SNP_1_168229057	C/T	1	168229057					−40.57	0.44		
	SNP_2_27401698	G/T	2	27401698					−37.77	0.31		
	SNP_2_46177221	G/A	2	46177221	147.63	2.52						
	SNP_6_104663091	A/C	6	104663091			115.82	9.30				
	SNP_6_124651063	G/A	6	124651063			−63.76	3.81				
	SNP_6_158401136	G/C	6	158401136			−72.23	3.41				
	SNP_7_173235732^#^	T/G	7	173235732	186.23	4.73						
	SNP_7_48994000	A/G	7	48994000	−166.31	5.17					
	SNP_8_38060255	T/C	8	38060255	−148.06	2.91					
	SNP_9_103241537^∗^	G/C	9	103241537	213.43	9.35	81.24	5.44				
	SNP_9_109684667^∗^	C/A	9	109684667					41.66	0.54	181.03	10.19
	SNP_10_143879663	G/A	10	143879663	163.20	4.53						
PT	SNP_2_79885513	T/C	2	79885513							0.31	10.34
	SNP_2_9506602^∗^	T/C	2	9506602			−0.18	5.14	−0.06	0.53	−0.24	9.74
	SNP_3_219463585	T/A	3	219463585			−0.10	4.67				
	SNP_4_211011498	T/C	4	211011498					−0.07	0.54		
	SNP_5_59630329	G/C	5	59630329					−0.07	0.58		
	SNP_8_22655499	T/A	8	22655499	0.34	5.00						
PTP	SNP_2_80464203	C/T	2	80464203	1.36	2.24						
	SNP_3_12888452^∗^	G/C	3	12888452			1.58	14.96	0.27	0.42		
	SNP_3_171824570^#^	G/T	3	171824570	−1.26	6.02						
	SNP_4_193530385	T/G	4	193530385	−1.36	3.71						
	SNP_4_71048778	A/G	4	71048778	−1.27	3.32						

*^#^Indicates the QTN identified across different traits. ^∗^Indicates the QTN identified across different methods.*

When comparing the results across different methods, only seven common QTNs are identified by more than one method simultaneously. Among these QTNs, SNP_2_9506602 is detected across three GWAS methods (FarmCPU, LASSO, and GEMMA); SNP_9_103241537 and one pleiotropic QTN (SNP_4_89429269) are identified by FASTmrEMMA and FarmCPU simultaneously; SNP_4_144401228 is detected by FASTmrEMMA and LASSO; SNP_9_109684667 is detected by LASSO and GEMMA; SNP_3_12888452 is detected by FarmCPU and LASSO. SNP_7_173235732 associated with SB and FV are detected by FASTmrEMMA and FarmCPU, respectively. Note that the estimated effects and *R*^2^ values (proportion of phenotypic variance explained by the QTL) of the co-identified QTNs detected by different methods are completely different, whereas the signs of effects for these co-identified QTNs for the same trait are consistent. For example, the estimated effects of SNP_2_9506602 are −0.177, −0.057, and −0.244, and the corresponding *R*^2^ values are 5.14, 0.53, and 9.74(%) for trait PT when using FarmCPU, LASSO, and GEMMA, respectively. All the three methods demonstrate that this QTN has the negative effect on PT.

### Simulation Studies for GWAS

Simulation experiments were performed to compare the statistical powers and Type I errors of the four GWAS methods. The statistical powers of detecting the simulated QTL calculated based on 200 simulations are given in **Table 4**. The average powers for FASTmrEMMA, FarmCPU, LASSO, and GEMMA were 55.19, 43.31, 53.69, and 40.44(%), respectively, indicating the highest average power of FASTmrEMMA. However, different methods may be suitable for detection of different QTL. For example, FASTmrEMMA has the highest power for detecting QTL1, QTL4, QTL5, QTL6, and QTL8 but the lowest power for detecting QTL7. LASSO is the best method for detecting QTL2 and QTL3, whereas it is the worst method for detecting QTL1. GEMMA has the lowest power of detecting all the simulated QTL, but it is the most efficient method for detecting QTL7. Type I errors for all the four methods are also listed in **Table 4**. LASSO has the lowest Type I error, followed by GEMMA and FASTmrEMMA, and FarmCPU has the highest Type I error. The Type I errors of the four methods are under 0.0001 with the same order of magnitude. Overall, the Type I errors are well controlled for all the four approaches, and the three multi-locus approaches are more powerful than the single-locus approach.

**Table 4:** Statistical powers (%) of eight simulated QTL and Type I error rates for four GWAS methods drawn from 200 replicated simulation experiments.

QTL	FASTmrEMMA	FarmCPU	LASSO	GEMMA
QTL1	52.5	22.5	5.5	6
QTL2	20.5	15.5	39	0
QTL3	55	46	62	41
QTL4	47	11.5	9	2.5
QTL5	92	61	83.5	60
QTL6	58	57	51	50
QTL7	20.5	65	92.5	94
QTL8	96	68	87	70
Type I error	6.99E-05	7.17E-05	4.70E-05	6.58E-05

## Discussion

In this study, we compared statistical powers of FASTmrEMMA, FarmCPU, LASSO, and GEMMA using real and simulation data. Simulation experiments based on the genotypic data of 230 maize inbred lines illustrate that the multi-locus approach is more powerful than single-locus approach in most cases, especially for loci with small effect that explain less than six percent of phenotypic variance. Although single-locus methods have been widely used to identify genetic variants in many crop species, they neglect the overall effects of multiple loci and suffer from the problem of multiple test corrections for critical values. Several investigators have compared statistic powers of multi-locus and single-locus methods and demonstrated that multi-locus methods perform better than single-locus methods. Wen et al. (2018) compared FASTmrEMMA with single-locus approaches including EMMA, SUPER, CMLM, and ECMLM using a series of simulation studies and found that FASTmrEMMA has the highest power and accuracy. Xu et al. (2017) showed that the multi-locus LASSO method has higher statistical power and lower Type I error than GEMMA. Liu X.L. et al. (2016) demonstrated that FarmCPU improves statistical power compared to GLM, MLM, CMLM, FaST-LMM-Select across multiple species, such as *Arabidopsis thaliana*, human and maize. In previous simulation studies, Bonferroni multiple test correction was used for single-locus method. However, it may be too strict to use Bonferroni correction (0.05/*m*) as the cut-off as not all loci are independent (Yang N. et al., 2014). To avoid missing the relevant loci, we replaced Bonferroni correction by a less stringent criterion (1/*m*) for GEMMA. The results of simulation showed that Type I error of GEMMA with 1/*m* as the cut-off was well controlled and similar to that of three multi-locus methods. Additionally, the permutation method is commonly used to adjust for multiple tests, which yields reliable outcome but requires a lot of time for huge samples (Churchill and Doerge, 1994). Fortunately, no multiple test correction is required for FASTmrEMMA and LASSO because all markers are fitted to a single model and all effects are estimated and tested simultaneously.

In the real data analysis, a total of 29, 19, 12, and 7 significant QTNs were identified for seven pasting properties of maize starch using FASTmrEMMA, FarmCPU, LASSO, and GEMMA, respectively. FASTmrEMMA detected the most QTNs, while GEMMA detected the least, which was consistent with the results of the simulation that FASTmrEMMA performed the best for detection of most QTL and GEMMA performed the worst. Unexpectedly, there was no significant QTN detected by these four methods simultaneously, and only seven QTNs were detected by more than one method. This situation could be explained by the simulation studies. From the simulation results, none of these methods were found to achieve very high power for detecting all the simulated QTL and different methods may be suitable for identification of different QTL. For example, FASTmrEMMA possessed good performance for most QTL, whereas it was not efficient for simulated QTL7 with the largest effect and lowest MAF. LASSO performed well for detecting large QTL but poorly for small QTL. Each method has its own advantages and limitations. LASSO is computationally efficient, but fails to handle a large number of markers. FASTmrEMMA is powerful in detection of QTL and accurate in effect estimation of QTL. However, FASTmrEMMA is a two-step combined method. The first step is to select a small fraction of makers and then apply these markers to perform multi-locus analyses in the second step. This method has an issue in how to determine the suitable thresholds in the first step. To improve the power of GWAS, it is better to use a combination of these methods, and the QTL detected by multiple methods may be more reliable. Recently, Zhang et al. (2018) and Ma et al. (2018) also proposed that using a combination of multiple multi-locus methods could improve the efficiency for detecting the QTL underlying lodging resistance-related and regeneration-related traits of maize.

Genome-wide association studies have been applied to dissect genetic architectures of several complex traits in maize (Xiao et al., 2017). However, no previous studies have focused on GWAS for starch pasting properties in maize. Here, we performed GWAS for seven pasting properties in a panel of 230 maize inbred lines genotyped with 145,232 SNPs and identified 60 significant QTNs using single-locus and multi-locus GWAS methods. Notably, the detected loci may not be the real causative loci due to false positives caused by LD or population structure. To understand the molecular basis of pasting properties, we further investigated locations of associated QTNs for possible candidate genes. The candidate genes within 250 kb downstream and upstream of the identified QTNs and their orthologs in Arabidopsis and rice are presented in **Supplementary Table S1**. According to functional annotations, these candidate genes were primarily categorized as protein kinases, glycosyltransferases, glycosidases, hydrolases, and transcription factors. The transcription factors included E2F, BHLH, TFIIH, MYB, bZIP, and HSF superfamily. Some of the candidate genes or their homologous genes are known genes linked to starch biosynthesis. For example, GRMZM2G032628 (*ae1*) encodes starch branching enzyme, which is a downstream gene involved in the final product of starch biosynthesis (Dolezal et al., 2014). It was reported that *ae1* was significantly associated with pasting properties of maize starch (Wilson et al., 2004). The homologous gene *SUS3* of GRMZM2G392988 in Arabidopsis has been reported to be involved in starch biosynthesis within seed coat and embryo (Angeles-Nunez and Tiessen, 2010). Several candidate genes are annotated as glycosyltransferases, which formed the important catalytic mechanism to synthesize and break the glycosidic bonds in oligosaccharides, disaccharides, and polysaccharides (Li et al., 2018). To better understand the potential biological functions of these candidate genes, we performed the gene ontology (GO) analysis for these genes using clusterProfiler (Yu et al., 2012). The GO analysis revealed that these genes were significant enriched in 16 GO terms (*P*-value <0.01), which were classified into three main types containing biological process, molecular function, and cellular component (**Supplementary Figure S1**). Under the first type, the most significant GO terms are gluconeogenesis process and hexose biosynthetic process, which play important roles in starch biosynthesis. Under the second type, these genes were significant related to chorismate synthase activity and glucose-6-phosphate isomerase activity. Under the third type, several genes were involved in photosystem. We also found that some candidate genes were involved in multiple functions. For example, GRMZM2G065083 are involved in gluconeogenesis process, hexose biosynthetic and metabolic process and glucose-6-phosphate isomerase activity. However, these genes were not found to be known genes involved in starch biosynthesis pathway, indicating that our study of the molecular mechanisms underlying pasting properties of maize starch is incomplete. These identified QTNs and candidate genes provide foundation for further functional studies to dissect the genetic mechanism manipulating maize pasting properties.

## Conclusion

In this study, single-locus and multi-locus GWAS methods were used to identify loci associated with starch pasting properties in maize. A total of 60 significant QTNs were detected for seven pasting properties, of which 29, 19, 12, and 7 QTNs were detected using FASTmrEMMA, FarmCPU, LASSO, and GEMMA, respectively. These QTNs could be utilized for further genetic and breeding studies to regulate starch pasting properties. Additionally, we compared four GWAS methods for their detection powers and Type I errors based on simulation studies and found that the multi-locus method is more powerful than the single-locus method and the combination of these multi-locus methods could help improve the statistical power of current GWAS.

## Author Contributions

CX and ZY designed the research plan. YX, TY, YZ, SY, PL, JL, and SX performed the experiments. YX and PL analyzed the data. YX wrote the paper. All authors read and approved the final manuscript.

## Conflict of Interest Statement

The authors declare that the research was conducted in the absence of any commercial or financial relationships that could be construed as a potential conflict of interest.

## Footnotes

1 https://github.com/BGI-shenzhen/PopLDdecay

2 http://maizegdb.org

3 https://phytozome.jgi.doe.gov

## Supplementary Material

FIGURE S1 Distribution of significant GO terms (*P*-value < 0.01).

TABLE S1 Candidate genes for QTNs significantly associated with starch pasting properties in maize and their homologous genes in Arabidopsis and rice.
