# Supplementary figures and images for: Genome-Wide Association Mapping of Starch Pasting Properties in Maize Using Single-Locus and Multi-Locus Models
Source: Front Plant Sci. 2018 Sep 5;9:1311. doi: 10.3389/fpls.2018.01311 (PMC6134291; doi:10.3389/fpls.2018.01311)

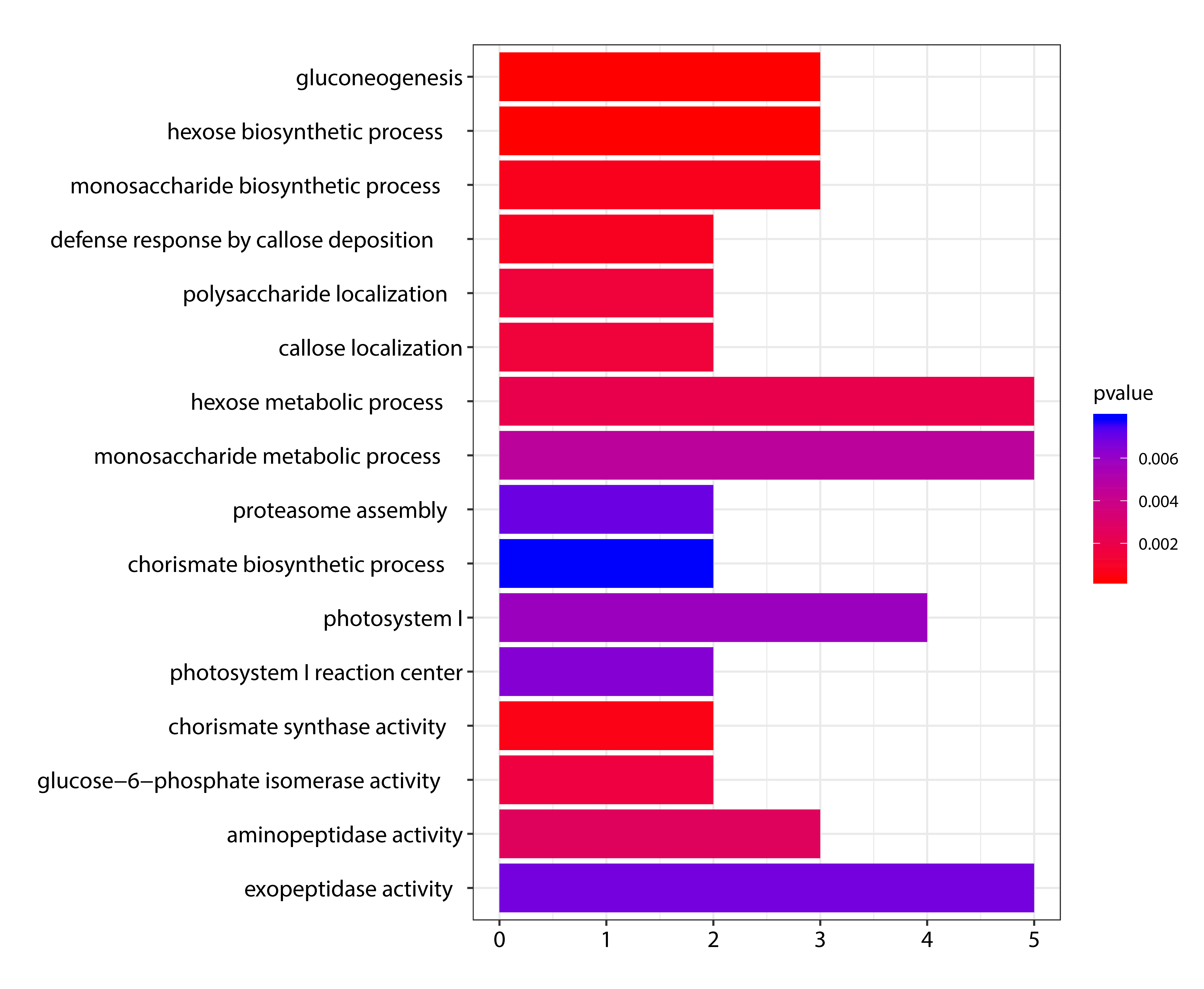

Supplement: FIGURE S1 — Distribution of significant GO terms (P-value < 0.01). [file Image_1.TIF]
